# Validation of the German capability for suicide questionnaire (GCSQ) in a high-risk sample of suicidal inpatients

**DOI:** 10.1186/s12888-020-02812-9

**Published:** 2020-08-20

**Authors:** Jan C. Cwik, Thomas Forkmann, Heide Glaesmer, Laura Paashaus, Antje Schönfelder, Dajana Rath, Sarah Prinz, Georg Juckel, Tobias Teismann

**Affiliations:** 1 Department of Clinical Psychology and Psychotherapy, Universität zu Köln, Cologne, Germany; 2 Department of Clinical Psychology, University of Duisburg-Essen, Essen, Germany; 3 Department of Medical Psychology and Medical Sociology, University Leipzig, Leipzig, Germany; 4 Mental Health Research and Treatment Center, Faculty of Psychology, Ruhr-Universität Bochum, Massenbergstrasse 11, 44787 Bochum, Germany; 5 Department of Psychiatry, LWL-University Hospital, Ruhr-Universität Bochum, Bochum, Germany

**Keywords:** Suicide, Fearlessness of death, Pain tolerance, Capability for suicid*e*

## Abstract

**Background:**

The German Capability for Suicide Questionnaire (GCSQ) was developed to measure fearlessness of death and pain tolerance – two constructs central to the Interpersonal Theory of Suicide. Initial scale development, definition of the factor structure and confirmation of the two-dimensional factor structure was performed in samples suffering from relatively low levels of suicide ideation/behavior. The present study aimed to validate the German Capability for Suicide Questionnaire (GCSQ) in a high-risk sample of suicidal inpatients.

**Methods:**

Factor structure, reliability and validity were investigated in a sample of inpatients (*N* = 296; 53.0% female; age in years: *M* = 36.81, *SD* = 14.27) admitted to a hospital due to a recent suicide attempt or an acute suicidal crisis (in immediate need of inpatient treatment). To establish convergent validity, interview-based assessments of lifetime suicide attempts and non-suicidal self-injury as well as questionnaire-based assessments of painful and provocative events were used. Finally, stability of GCSQ-scores over a follow-up period of 12 months was assessed.

**Results:**

Results indicated good psychometric properties, and provided additional evidence for construct validity and stability of the subscales over a one-year period, and demonstrated adequate fit of the data with respect to the original factor structure.

**Conclusions:**

Results suggest that the GCSQ is a brief, reliable, and valid measure of capability for suicide that can be used in clinic assessment and research.

## Background

The capability to die by suicide, that is, the ability to endure the dread and pain associated with suicide, is a construct central to the understanding of suicidal behavior [1]. Three major theories of suicidal behavior – the Interpersonal Theory of Suicide [IPT [2];], the Integrated Motivational-Volitional (IMV) Model of Suicidal Behaviour [3] and the Three-Step Theory [3-ST [4];] concur on the importance of fearlessness about death and pain tolerance as a differentiating factor between individuals thinking about suicide and individuals attempting suicide or dying by suicide. These so-called ideation-to-action theories [5] agree that suicide ideation is unlikely to result in suicidal behavior if a person is lacking capability for suicide. Within the framework of the IPT, Joiner [2] proposed that engaging in suicidal behavior (e.g., suicide attempts) will most likely lead to acquired capability for suicide. However, a person can also become desensitized to the fear and pain associated with suicide through other painful and provocative events (e.g., combat exposure, childhood abuse). Shifting from the idea that capability for suicide is solely acquired, Klonsky and May [4] recently suggested a broader concept that also considers potential genetic (*dispositional capacity*) or situational factors (*practical capacity*). Therefore, the original term acquired capability for suicide has been replaced by the more broad term capability for suicide [6].

Evidence regarding the central role of capability for suicidal behavior is mixed: some studies have shown that fearlessness about death differentiates between suicide ideators and suicide attempters [4, 7–9], whereas others found no differences between suicide attempters and ideators or non-ideators with respect to fearlessness about death [10, 11] or pain tolerance [12]. Furthermore, findings on the relevance of an interaction between suicide ideation (or risk factors for suicide ideation) and capability for suicide in predicting suicidal behavior are inconclusive [6, 13]: In a systematic review, Ma et al. [13], found only three (42.8%) out of seven tests on the interaction between two risk factors for suicide ideation (i.e., thwarted belongingness and perceived burdensomeness) and capability for suicide to predict suicide attempt status. Finally, some studies challenge the idea that experiencing painful and provocative events results in a prospective increase in capability for suicide [14]; instead capability for suicide has been shown to fluctuate when assessed on a daily basis [15] and to be rather static when assessed over longer time periods [14, 16]. Although the relevance of capability of suicide is unquestioned [1], the associations involved might be more complex [17].

To assess an individual’s extent of acquired capability, Joiner and colleagues [18] developed the Acquired Capability for Suicide Scale (ACSS) containing 20 items. Ribeiro and colleagues [19] revised the ACSS and presented the Fearlessness about Death-Scale (ACSS-FAD) consisting of seven items assessing solely fearlessness about death. The scale has adequate to good internal consistency (α ranging from 0.77 to 0.85) as well as good construct validity. Overall, the scale proved to be a viable measure of an individual’s extent of fearlessness of death [20]. However, the scale does not assess a person’s pain tolerance. To facilitate the assessment of capability for suicide and its two facets, a German questionnaire, which was based on the original ACSS and assesses both fearlessness of death and dying as well as pain tolerance was developed: the German Capability of Suicide Questionnaire (GCSQ [21];). A validation study was carried out with two clinical (*n* = 424) and an online sample (*n* = 532). Exploratory and confirmatory factor analysis showed that the questionnaire indeed comprises two factors: fear about death (GCSQ-FAD scale) and pain tolerance (GCSQ-PT scale). Furthermore, an item assessing the individual’s perceived capability for suicide was retained in the questionnaire (“perceived capability”-item; GCSQ-PC), as it has shown to predict the individual’s suicide attempt history (cf. [12, 22]). The scales and the “perceived capability”-item displayed good construct validity correlating in the expected direction with relevant measures such as pain sensitivity, acceptance of one’s own dying and death, impulsivity and sensation seeking. Furthermore, the GCSQ-pain tolerance scale was shown to predict heightened objective pain tolerance as assessed with a cold-pressure task, as well as risk-taking behavior within a gambling task [23].

Questionnaire development is an iterative process over several stages. Though an inpatient sample (*n* = 244) was used in the original validation study [21], only 30.6% of the sample had attempted suicide during their lifetime. Hence, it is important to evaluate the psychometric properties and factor structure of the GCSQ in a high-risk sample of suicidal inpatients (cf. [6]). The current study therefore administered the GCSQ in a sample of inpatients admitted to a psychiatric hospital due to severe suicide ideation or a recent suicide attempt and included measures for additional evaluations of construct validity: To assess lifetime suicide attempts and non-suicidal self-injury, the revised form of the Painful and Provocative Events Scale (PPES [24];) and the Self-Injurious Thoughts and Behavior Interview (SITBI [25];) were included. Finally, stability of GCSQ-scores was assessed within a follow-up period of 6, 9 and 12 months.

## Methods

### Participants and procedure

The current study is part of a multicenter study on risk factors for suicide attempts called “Predictors of suicidal ideation and suicidal behavior in a high-risk sample (PRESS)” (see also [12]). The total sample comprised 308 inpatients (53.6% female; age: *M* = 36.92, *SD* = 14.30) admitted to a hospital due to attempted suicide or an acute suicidal crisis (in immediate need of inpatient treatment). Data on the GCSQ were available for 296 inpatients (53.0% female; age in years: *M* = 36.81, *SD* = 14.27). The most common primary diagnoses according to the International Classification of Diseases (ICD-10) were affective disorders (*n* = 228; 77.0%), mental and behavioral disorders due to psychoactive substance use (*n* = 56; 18.9%) as well as neurotic, stress-related and somatoform disorders (*n* = 106; 35.8%). One hundred and ninety-one (64.54%) patients reported at least one lifetime suicide attempt.

Data were collected in 13 psychiatric hospitals in Germany. If patients agreed to participate, the Self-Injurious Thoughts and Behaviors Interview (SITBI [25];) and the “Diagnostisches Interview bei psychischen Störungen – Short version” (MINI-DIPS), a structured clinical interview with well-established reliability, validity, and patient acceptance [26] were conducted by trained clinical psychologists. Additional questionnaires were presented in a paper-pencil version. Psychiatric inpatients were interviewed at the latest 14 days after admission to a psychiatric ward (T0) and again after six (T1), nine (T2) and 12 months (T3). The T1 and T2 assessments were conducted by phone, with questionnaires sent by mail; the T3 assessment was conducted at one of the participating treatment centers. The vast majority of assessments took place within a period of ten to 14 days of the reference day. Participants who took part in all four assessments (*n* = 118) did not differ from other participants regarding age, gender, lifetime number of non-suicidal self-injury and painful and provocative events. However, they did differ regarding lifetime number of suicide attempts, *t*(290) = 2.55, *p* = .01. As such, participants who took part in all four assessments had attempted suicide less frequently in their lifetime than participants who did not take part in all four assessments.

All participants were informed about the aim of the study, the voluntary nature of their participation, data storage and data security. All participants gave written informed consent before partaking. The study was approved by the responsible Ethics Committees (EK 310/13, Medical Faculty of RWTH Aachen University; 4909-14, Medical Faculty of the Ruhr University Bochum; 042-14-27,012,014, Medical Faculty of the University of Leipzig).

### Measures

#### *German Capability for Suicide Questionnaire* (*GCSQ* [21, 23];)

The GCSQ is an 11-item self-report measure comprising two scales: one scale assesses fearlessness about death (GCSQ-FAD) with five items (e.g., “I am very much afraid to die”), and the other scale assesses subjective pain tolerance (GCSQ-PT) with five items (e.g., “When in pain, I clench my teeth and carry on.”). Furthermore, the GCSQ contains a perceived capability item (GCSQ-PC: “I could kill myself, if I wanted to.”). Items are answered on a scale ranging from 1 (“*I fully agree”*) to 5 (“*I do not agree at all”*). Higher scores indicate greater fearlessness and/or pain tolerance.

#### *Painful and Provocative Events Scale-German* (*PPES-G* [24];)

The German version of the PPES comprises two subscales assessing lifetime experiences with *passive* painful and provocative events (i.e., physical and sexual abuse) with 4 items and *active* painful and provocative events (e.g., rock climbing, gun shooting) with 8 items. Items are rated on a 5-point Likert scale ranging from 1 (“*never”*) to 5 (“*more often than 20”).* Internal consistency was shown to be modest (α ≥ 0.63) in the validation study [24], as well as in the current sample: α = 0.69 for passive PPE and α = 0.67 for active PPE. Low construct reliabilities have repeatedly been reported for the PPES [19], which is commonly explained by the fact that the items in a life event measure are largely independent events and thus do not necessarily share much common variance [19].

#### *Self-Injurious Thoughts and Behaviors Interview* (SITBI [25];)

The SITBI is a structured interview that assesses the presence, frequency, and characteristics of a wide range of self-injurious thoughts and behaviors. Within the current analysis, the following items were used to assess a lifetime history of suicide attempts: “How many suicide attempts have you made in your lifetime?” (Item 40) and “How many times in your life have you engaged in NSSI” (Item 65). Interrater- and retest-reliability as well as convergent validity of the German version of the SITBI [27] have been shown to be adequate.

### Statistical analyses

First, an item analysis was conducted to investigate the means, standard deviations, skewness, kurtosis, and corrected item-total correlation of the GCSQ in a high-risk sample. A corrected item-total correlation of > .30 indicates an acceptable correlation [28]. Additionally, to test whether items of the GCSQ are useful to measure variability in the capability for suicide and to investigate whether the items are too sensitive (easy item) or too specific (hard item), the item difficulty was investigated. Participants tend to rate “easy items” more likely higher than items with a moderate item difficulty, so that very easy items are too sensitive compared with other items of the scale: Most of the participants would be identified as potentially having a higher fearlessness about death or higher pain tolerance. Contrarily, participants rate hard items more likely with lower scores compared with moderate items. Thus, such items could identify most participants as having a lower fearlessness about death or lower pain tolerance. An item difficulty index of > 85% indicates an easy item, whereas an item difficulty index of between 51 and 84% indicates a moderate item, and hard items have an item difficulty index of < 51%. An ideal difficulty index is about 70% for 5-point Likert scales [29].

In order to test the original two-factor structure of the GCSQ [21] in a high-risk sample, a confirmatory factor analysis (CFA) was conducted. Considering the skewness and kurtosis of the items as well as the ordinally distributed data, a weighted least squares-mean robust estimator (WLSM) was used. For comparison, the one-factor model was also tested. To estimate the model fit of the one-factor and the two-factor solution of the GCSQ, the following goodness of fit indices were calculated: the relative χ^2^ (χ^2^/*df*), the root-mean-square-error-of-approximation (RMSEA) including the 90% confidence interval (90%-CI), the comparative-fit-index (CFI), the Tucker–Lewis index (TLI), and the standardized root-mean-square residual (SRMR). A relative χ^2^ of < 3 indicates a good model fit [30]. A CFI and a TLI of > 0.90 are indicators of an adequate fit, whereas values > 0.95 indicate a good fit [30, 31]. For the RMSEA, values of < 0.05 indicate a good model fit, whereas values between < 0.08 and > 0.05 can be seen as reasonable fit [32]. In case of the SRMR, values of < 0.09 represent a good model fit [33].

McDonald’s ω [34–36] was calculated for the subscales of the GCSQ to investigate the internal consistencies, because structural equation modeling based methods are relatively robust regarding biases due to low numbers of items [37, 38]. To receive a comprehensive picture of the internal consistencies of both subscales, ω was calculated for each subscale and each measurement point (T0-T3). The internal consistency is interpreted as follows: ω < 0.50: unacceptable; ω < 0.60: poor; ω < 0.70: questionable; ω < 0.80: acceptable; ω < 0.90: good; ω ≥ 0.90: excellent.

Test-retest-reliability over the four measurement time points (T0-T3) was investigated by using the measurement of the mean of four measurements intraclass correlation coefficient (ICC) with the 95%-confidence interval (95%-*CI*), by using a two-way random-effects model. ICC is interpret as follows: < 0.50: poor, 0.50 - ≤ 0.75: moderate, 0.75 - ≤ 0.90: good, and > 0.90: excellent [39].

The distribution of data was investigated by using the Kolmogorov-Smirnov-test. Due to the ordinal distribution of data, non-parametric methods were used to analyze the data. For all analyses, a pairwise deletion of cases with missing data was used. For the investigation of the convergent validity, the associations between GCSQ scores and criterion measures were analyzed with Spearman’s rank-correlation coefficients (*r*_*S*_).

## Results

### Item analysis and item-total correlation

The item analysis showed that participants in this study reported a medium manifestation of capability for suicide, due to the fact that the means of items ranged from 2.887 – 3.993 (see Table 1). Item difficulties showed a moderate index for all items, with an ideal item difficulty index of about 70% for most of the items. Additionally, all items showed acceptable corrected item-total correlations of > .388.

**Table 1:** Results of the item analysis and the item-total correlation analysis of the German Capability for Suicide Questionnaire

Item number	*M*	*SD*	Range	Skewness	Kurtosis	Item difficulty	Corrected item-total correlation
1	2.976	1.511	1-5	0.029	−1.444	59.52	0.525
2	2.887	1.554	1-5	0.183	−1.513	57.74	0.606
3	3.415	1.470	1-5	−0.387	−1.312	68.30	0.826
4	3.412	1.473	1-5	−0.391	−1.294	68.24	0.825
5	3.461	1.495	1-5	−0.425	−1.298	69.22	0.715
6	3.993	1.248	1-5	−1.145	0.240	79.86	–
7	3.399	1.132	1-5	−0.435	−0.901	67.98	0.413
8	3.276	1.398	1-5	−0.245	−1.241	65.52	0.367
9	3.584	1.249	1-5	−0.530	−0.789	71.68	0.695
10	3.646	1.341	1-5	−0.613	−0.857	72.92	0.590
11	3.835	1.207	1-5	−0.946	−0.047	76.70	0.388

Note: Corrected item-total correlations for items 1-5 were calculated for the GCSQ-FAD factor, and for items 7-11 for the GCSQ-PT factor

### Confirmatory factor analysis

**Fig. 1:**
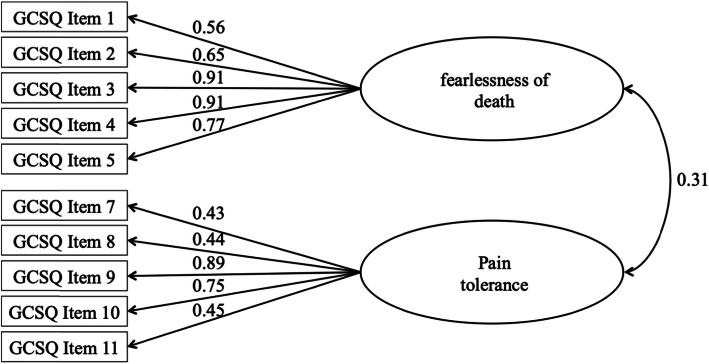
Structural equation model with pathways between the two factors of the German Capability for Suicide Questionnaire

The CFA for the one-factor solution of the GCSQ showed an insufficient model fit: relative χ^2^ = 15.46; CFI = 0.84, TLI = 0.79, RMSEA = 0.162 (90%-CI: 0.150 - 0.174), and SRMR = 0.139. In contrast, the CFA showed an overall excellent model fit for the two-factor solution of the GCSQ (see Fig. 1). The CFA revealed a relative χ^2^ of 1.88, which is an indicator of a good model fit. In addition, the other indicators indicate an excellent model fit for the two-factor solution of the GCSQ: CFI = 0.99, TLI = 0.98, RMSEA = 0.041 (90%-CI: 0.025 - 0.056), and SRMR = 0.049. Furthermore, all items showed medium to high standardized factor loadings on the assigned factors, ranging from 0.45 – 0.91.

### Internal consistency

The internal consistency of subscales of the GCSQ was investigated by calculating ω for all four measurement points (T0-T3). The results revealed a good internal consistency of the GCSQ-FAD for T0 with ω = 0.88 (*n* = 295) and for T1 with ω = 0.87 (*n* = 170) and an excellent internal consistency for T2 with ω = 0.91 (*n* = 165) and for T3 with ω = 0.90 (*n* = 150). The GCSQ-PT subscale revealed an acceptable internal consistency at all four measurement points (T0: ω = 0.74, *n* = 296; T1: ω = 0.73, *n* = 169; T2: ω = 0.78, *n* = 165; T3: ω = 0.70, *n* = 150). Thus, the internal consistency of both subscales, measured in a high-risk inpatients sample, is in a comparable range as reported by Wachtel et al. [21].

### Test-retest-reliability

The test-retest-reliability was investigated for both subscales of the GCSQ and the GCSQ-PC item in a subsample of *n* = 118 (38.6%) participants who took part in all four measurement time points (T0-T3). The investigation of the ICC across all four measurement time points for this subsample revealed an excellent test-retest-reliability (ICC: .94 [95%-*CI*: .92 - .96]) for the GCSQ. An ICC of 0.92 (95%-*CI*: 0.90 - 0.94) for the GCSQ-FAD and an ICC of 0.92 (95%-*CI*: 0.90 - 0.94) for the GCSQ-PT subscales indicate an excellent test-retest-reliability of both subscales of the GCSQ. For the GCSQ-PC item, an ICC of 0.78 (95%-*CI*: 0.70 - 0.84) was observed, which indicates a good test-retest-reliability of this item.

### Convergent validity

As illustrated in Table 2, the fearlessness about death subscale of the GCSQ (GCSQ-FAD), the pain tolerance subscale of the GCSQ (GCSQ-PT), and the perceived capability item of the GCSQ (GCSQ-PC) showed significant intercorrelations.

**Table 2:** Results of Spearman’s rank correlation analyses

	GCSQ-PT	GCSQ-PC	PPES passive	PPES active	Number of lifetime suicide attempts	Number of lifetime NSSI
GCSQ-FAD	0.234*** *n* = 295	0.235*** *n* = 293	0.166** *n* = 292	0.149* *n* = 294	0.067 *n* = 291	0.128* *n* = 288
GCSQ-PT	–	0.143* *n* = 294	0.105 *n* = 294	0.182** *n* = 296	−0.029 *n* = 293	−0.032 *n* = 290
GCSQ-PC		–	0.221*** *n* = 291	0.070 *n* = 293	0.174** *n* = 290	0.141* *n* = 287

Note: *NSSI* Non-suicidal self-injuries, *GCSQ* German Capability for Suicide Questionnaire, *GCSQ-FAD* Fearlessness about death subscale, *GCSQ-PT* Pain tolerance subscale, *GCSQ-PC* Perceived capability item, *PPES* Painful and Provocative Events Scale, * = *p* < 0.05; ** = *p* < 0.01; *** = *p* < 0.001

Table 2 additionally shows that higher scores in the GCSQ-FAD subscale are significantly associated with higher scores in the PPES passive subscale, the PPES active subscale, and more lifetime NSSI measured with the SITBI. The GCSQ-PT subscale showed a significant positive association with the PPES active subscale. Furthermore, the higher scores at the GCSQ-PC item were significantly associated with higher scores in the PPES passive subscale, and with more lifetime suicide attempts as well as with more lifetime NSSI measured with the SITBI.

## Discussion

The current study provides additional evidence for the psychometric properties of the GCSQ using a high-risk sample of suicidal inpatients. Importantly, this study offers support for the 2-factor structure - with one factor “Fearlessness of Death” and the second factor “Pain Tolerance” - in a sample that is more representative of the scale’s target population than the samples previously investigated [21, 23]. The subscales demonstrated moderate to good internal consistencies and acceptable item-total correlations. Also, the subscales and the “perceived capability”-item correlated, which underlines that the facets of capability for suicide are not fully independent of each other. Furthermore, GCSQ-scores were shown to be stable over a follow-up period of 12 months, supporting the trait-like character of capability for suicide (cf. [14, 16]).

This study also provides additional evidence for the construct validity of the GCSQ. As such, it was shown that “Fearlessness of death” and “Perceived capability” were associated with lifetime number of non-suicidal self-injury and the “Perceived capability” was shown to be associated with lifetime number of suicide attempts. Active painful and provocative events (e.g., rock climbing, gun shooting) were associated with “Fearlessness of Death” and “Pain Tolerance”; whereas passive painful and provocative events (i.e., childhood trauma), were associated with “Fearlessness of Death” and “Perceived capability”. The rather modest size of all correlations is in line with previous findings [19, 21, 24] and underlines the necessity to not perceive capability of suicide exclusively as a product of previous painful and provocative events or self-injurious behavior (cf. [4]). In addition, the pattern of findings refers to the differential associations of the various facets of capability and thus to the necessity of covering capability of suicide broadly and not limiting it to one facet (cf. [40]).

The role of pain tolerance, however, remains unclear: First of all, internal consistencies of the scale are remarkably lower than for the GCSQ-FAD scale, and in addition, the GCSQ-PT scale is hardly associated with relevant markers of suicide risk (cf. [21]). Although Wachtel et al. [23] found an association between GCSQ-PT scores and objective pain tolerance (assessed with a cold-pressor task), this finding was not replicated by Paashaus et al. [12] who assessed pain tolerance with a pressure algometer. Furthermore, using the current study sample, Paashaus et al. [12] found no differences in GCSQ-PT scores between suicide ideators and suicide attempters (the same being true for GCSQ-FAD scores). In general, it seems to be difficult to capture the concept of insensitivity to pain in self-report [41], so it may be appropriate to measure pain tolerance using objective pain tests whenever possible (cf. [42]). However, only certain quality of pain can be induced by specific pain induction methods, therefore, a comprehensive assessment of self-reported pain tolerance cannot be completely avoided.

The current study complements previous studies emphasizing the importance of “Perceived capability”: An individual’s perception of one’s own capability to attempt suicide significantly predicted attempt status and the occurrence of suicidal behaviors in previous studies [21, 43] and differentiated between those who had attempted suicide from those who had not in the current study sample [12] as well as in an Australian student sample [22]. Therefore, it seems reasonable and important to ask that very question in a clinical context: “I could kill myself if I wanted to ­ how strongly do you agree with that statement?”

Several limitations have to be considered when interpreting the results of the present study. First of all, the GCSQ was not compared to existing measures of capability of suicide. Comparisons with the Acquired Capability with Rehearsal for Suicide Scale (ACWRSS [40];) and the Suicide Capacity Scale (SCS-3 [4];) would be of particular interest. The ACWRSS is a 7-item questionnaire based on the traditional idea that capability is acquired through preparation behavior, whereas the SCS-3 is a 6-item measure assessing dispositional, acquired and practical capability for suicide. The GCSQ-FAD scale shares most items with the ACSS-FAD presented by Ribeiro and colleagues [19], therefore comparisons with the ACSS-FAD seem of less interest. Second, the current study lacks an analysis of the predictive abilities of the GCSQ to prospectively predict suicide attempts; however – using the current study sample – Forkmann et al. (Forkmann T, Glaesmer H, Paashaus L, Rath D, Schönfelder A, Juckel G, Assion J, Stengler K, Teismann T: Interpersonal-psychological theory of suicide: a prospective examination, submitted) found no indication that capability for suicide is predictive of future suicide attempts. Prospective studies using different measures to assess capability for suicide have to be awaited before determining whether methodological or theoretical inaccuracies underlie this finding. Nevertheless, the central importance of the capability construct for understanding suicidal behavior is strongly questioned by current evidence (cf. [6, 12, 13] (Forkmann T, Glaesmer H, Paashaus L, Rath D, Schönfelder A, Juckel G, Assion J, Stengler K, Teismann T: Interpersonal-psychological theory of suicide: a prospective examination, submitted)). This is all the more true since two recent studies have comprehensively shown that no simple algorithm (i.e., a single factor such as capability for suicide) can accurately distinguish between suicide attempters and suicide ideators [44] or between (lifetime) suicide attempters and individuals engaging in non-suicidal self-injury [45]. These findings imply that recent ideation-to-action models do not sufficiently take into account the complexity of suicidal behaviour.

## Conclusion

In summary, the current results suggest that the GCSQ is a brief, reliable, and valid measure of capability for suicide that can be used in clinic and research settings.

## Data Availability

All relevant data are reported within the paper. Analyzed data are available from the corresponding author on reasonable request. English language versions of all questionnaires used in the current study have already been published. The corresponding citations can be found below.

## Abbreviations

ACSS: Acquired Capability for Suicide Scale
ACSS-FAD: Acquired Capability for Suicide Scale - Fearlessness about Death-Scale
ACWRSS: Acquired Capability with Rehearsal for Suicide Scale
CFA: Confirmatory factor analysis
CFI: Comparative-fit-index
GCSQ: German Capability for Suicide Questionnaire
GCSQ-FAD: German Capability for Suicide Questionnaire - Fearlessness about Death-Scale
GCSQ-PT: German Capability for Suicide Questionnaire – Pain tolerance scale
GCSQ-PC: German Capability for Suicide Questionnaire – Perceived Capability
ICC: Intraclass correlation coefficient
IMV: Integrated Motivational-Volitional Model of Suicidal Behaviour
IPT: Interpersonal Theory of Suicide
MINI-DIPS: Diagnostisches Interview bei psychischen Störungen – Short version
NSSI: Non-suicidal self-injury
PPES: Painful and Provocative Events Scale
RMSEA: Root-mean-square-error-of-approximation
SITBI: Self-Injurious Thoughts and Behavior Interview
SRMR: Standardized root-mean-square residual
TLI: Tucker–Lewis index
WLSM: Weighted least squares-mean robust estimator
